# Circulation of Crimean-Congo Hemorrhagic Fever Virus in the Former Yugoslav Republic of Macedonia Revealed by Screening of Cattle Sera Using a Novel Enzyme-linked Immunosorbent Assay

**DOI:** 10.1371/journal.pntd.0003519

**Published:** 2015-03-05

**Authors:** Marc Mertens, Zati Vatansever, Slavcho Mrenoshki, Kiril Krstevski, Jovana Stefanovska, Igor Djadjovski, Iskra Cvetkovikj, Robert Farkas, Isolde Schuster, Fabien Donnet, Loic Comtet, Noël Tordo, Mohamed Ben Mechlia, Anne Balkema-Buschmann, Dine Mitrov, Martin H. Groschup

**Affiliations:** 1 Institute of Novel and Emerging Infectious Diseases, Friedrich-Loeffler-Institut, Federal Research Institute for Animal Health, Greifswald—Isle of Riems, Germany; 2 Faculty of Veterinary Medicine, Kafkas University, Department of Parasitology, Kars, Turkey; 3 Faculty of Veterinary Medicine, Saints Cyril and Methodius University, Skopje, Macedonia; 4 Faculty of Veterinary Science, Szent István University, Budapest, Hungary; 5 IDVet, Grabels, France; 6 Unit Antiviral Strategies, Institut Pasteur, Paris, France; Stanford University School of Medicine, UNITED STATES

## Abstract

**Background:**

There are only few assays available for the detection of Crimean-Congo Hemorrhagic Fever Virus (CCHFV)-specific antibodies in animals, and data about diagnostic sensitivity and specificity are incompletely documented for most of these tests. This is unfortunate since CCHFV antibodies in animals can be used as indicator for virus circulation in a geographic area and therewith potential risk of human exposure. This paper therefore reports on a novel ELISA for the detection of CCHFV-specific antibodies in cattle and on its application for testing ruminant sera from the Former Yugoslav Republic of Macedonia.

**Principal Findings:**

A highly sensitive and specific ELISA was developed to detect CCHFV-specific IgG antibodies in cattle. The assay was validated by using 503 negative serum samples from a country where CCHFV has never been detected until now, and by using 54 positive serum samples. The positive sera were verified by using two commercially available assays (for testing human serum) which we have adapted for use in animals. The sensitivity of the novel ELISA was 98% and its specificity 99%. The presence of Hyalomma ticks was demonstrated in the Former Yugoslav Republic of Macedonia and depending on the region antibody prevalence rates up to 80% were detected in the cattle population.

**Conclusion:**

This article describes a fully validated, highly sensitive and specific ELISA for the detection of CCHFV-specific IgG antibodies in cattle. Using this assay, CCHFV-specific antibodies were detected for the first time in cattle in the Former Yugoslav Republic of Macedonia, giving evidence for an active circulation of this virus in the country. Supporting this conclusion, the occurrence of the main vector of CCHFV was demonstrated in the present work for the first time in Former Yugoslav Republic of Macedonia.

## Author Summary

Crimean-Congo hemorrhagic fever virus (CCHFV) infections in humans can cause a deadly hemorrhagic fever with high fatality rates. Most humans are infected by ticks or by contact with blood. No vaccine prophylaxis or therapeutic interventions are available. Ticks of the genus *Hyalomma* function as vector of CCHFV. Domestic ruminants play a crucial role in the transmission and amplification of the virus. Since infected ruminants do not develop clinical signs, CCHFV infections can in most cases only be detected by serology. There are only few assays available for the detection of CCHFV-specific antibodies in animals. In general, the real distribution of CCHFV is fairly unknown. Here we present a novel, highly sensitive and specific ELISA, and the adaptations for two commercially available assays for the detection of CCHFV-specific antibodies in cattle. With these assays, we detected CCHFV-specific antibodies in cattle in the Former Yugoslav Republic of Macedonia. This is the first indication of an active circulation of CCHFV in this country. This knowledge will assist decision makers and public health authorities in deciding on effective countermeasures and can help to prevent outbreaks.

## Introduction

Crimean-Congo hemorrhagic fever virus (CCHFV) is a member of the genus *Nairovirus* in the family *Bunyaviridae* and belongs to the Arboviruses (**Ar**thropod-**bo**rne viruses).CCHFV is transmitted primarily by ticks belonging to the genus *Hyalomma* which function as vector as well as natural reservoir [1]. *Hyalomma marginatum* ticks have been found in many European countries south of the 46^th^ latitude [2,3]. The virus circulates in a tick-vertebrate-tick cycle, but it can also be transmitted by co-feeding, horizontally (transstadial) and vertically (transovarial) in the tick population [4,5]. Depending on their stage of maturity, Hyalomma ticks infest a wide spectrum of wildlife (e.g. hedgehogs, ground-feeding birds and hares) and domestic animals (e.g. goat, cattle and sheep) which play a crucial role in their life cycle and in the amplification and spread of the virus [6]. The viremia can last for up to two weeks in vertebrates. Although a seroconversion can be detected, none of the aforementioned species seem to develop clinical signs following a CCHFV infection [7]. In contrast CCHFV infections in humans can cause a deadly hemorrhagic fever. Human cases have been reported from more than 30 countries of Asia, South-Eastern Europe and Africa [1]. In Turkey, more than 1,000 human CCHF cases were reported annually in some years of the last decade [8]. In Europe, human cases occur regularly in Albania, Bulgaria and Kosovo, while infection rates and case numbers in most other countries are fairly unknown [9]. Case fatality rates of 5% (in Turkey) to 80% (in China) have been reported and may depend on the virus strain, education and awareness of individuals and communities as well as on the effectiveness of the public health system [9]. Most humans acquire the infection by tick bites and by crushing infected ticks, but infections are also possible through contact with blood and other body fluids of viremic animals. Moreover, CCHFV can be transmitted directly from human-to-human, demonstrated by a lot of nosocomial outbreaks (accidents, surgeries, unprotected contact to blood of patients etc.) [10–12]. Currently, there is no CCHF vaccine available and the therapy is restricted to symptomatic treatment [13].

Serological screening of ruminants allows the identification of geographical areas in which CCHFV circulates. Seroprevalence rates in domestic animals of up to 79% were reported in the endemic areas in Turkey [14]. Apart from countries with notified human CCHF cases, data on infections in animals are rare. The consequence is that the real prevalence in animals and the distribution of CCHFV in Europe and elsewhere is largely uncertain. However, only few assays for the detection of CCHFV-specific antibodies in animals have been published and information regarding diagnostic sensitivity and specificity for these are frequently incomplete [9].

The presence of CCHFV in Southern Europe and the risk of a northerly spread of the virus is a challenge for the European Union. Surveillance programs in humans, domestic animals and in ticks should be implemented in countries with suitable habitats for vector competent ticks [9,15].

In the present work, a seroepidemiological study was conducted among bovines to investigate a possible circulation of CCHFV in the Former Yugoslav Republic of Macedonia. For this purpose a novel ELISA was developed and validated, as well as two commercially available assays were adapted for the detection of CCHFV-specific antibodies in cattle.

## Materials and Methods

### Ethics statement

Serum collections were carried out in compliance with fundamental ethical principles for diagnostic purposes in the context of national surveillance studies. Serum samples from Macedonia were taken for serological testing of bovine brucellosis. This is an activity performed under the national multi-annual Program for control and eradication of bovine brucellosis official documented and supported by the Macedonian Government/FVA. The Program is designed according to the Council Directive 64/432/EEC of 26 June 1964 on animal health problems affecting intra-Community trade in bovine animals and swine. The serum samples from Turkey were collected as a part of the CCHFV surveillance referring to the “Protocol on risk assessment on CCHF 12.9.2011” (signed between Ministry of Health (MoH) and Ministry of Forestry and water affairs) and the “Decisions on the epidemiological surveillance of CCHF made by MoH in accordance to CCHF advisory board suggestions” (28.11.2006, 3.1.2013, 6.3.2014). The serum samples from Germany were collected from clinically healthy animals on the order of the national veterinary services in the context of herd slaughter practices after a notifiable disease outbreak. The Friedrich-Loeffler-Institut (FLI), Federal Research Institute for Animal Health, is an independent higher federal authority under the Federal Ministry for Food and Agriculture.

### CCHFV reference sera

102 serum samples from cattle from endemic areas in Turkey were collected in 2011 as a reference panel for the development and validation of the new ELISA. All sera were tested under suitable biosafety conditions by quantitative RT-PCR to exclude the presence of CCHFV prior to their serological characterization. The sera were tested for CCHFV-specific antibodies using an adapted commercially available ELISA (Vector-Best, Novosibirsk, Russia) (see below). Positive serum samples were subsequently confirmed by an adapted immunofluorescence assay (IFA; Euroimmun, Lübeck, Germany) (see below). Only sera which were positive in both assays were included as positive control sera in the reference panel. As negative control samples, 503 sera from cattle from Germany, a country currently free of CCHFV, were used.

### Serum and tick samples from the Former Yugoslav Republic of Macedonia

For the seroepidemiological study, 158 serum samples from cattle from Former Yugoslav Republic of Macedonia were collected between 2009 and 2011. Sera originated from the Northeastern region (n = 20), from the Skopje region (n = 35), from the Vardar region (n = 23), and from the Southeastern region (n = 80). Dry regions with grass and bush land were selected for the collection of serum samples and ticks, since those are preferred habitats of the vectors for CCHFV. The samples were collected at different farms in each region. The ticks were collected from goat, bovine and sheep in May and June 2012.

### Adaptation of a commercial CCHFV IgG-ELISA

A commercial ELISA for the detection of CCHFV-specific antibodies in human sera (Vector-Best) was adapted for use in cattle sera. All washing steps were performed with PBS-Tween. Bovine sera were diluted 1:100 in dilution buffer (90% SDB- and 10% SPSD-buffer of the manufacturer). The serum dilutions (100 μl/well) were incubated for 1 h at 37°C. After washing the plates, 100 μl/well of goat anti-bovine IgG-HRP conjugate (Southern Biotech) diluted 1:6.000 in conjugate dilution buffer (by manufacturer) was added and incubated for 30 min at 37°C. After washing the plates, 100 μl/well Tetramethylbenzidine (TMB, Bio-Rad, Munich, Germany) solution was added, and the reaction was stopped with H_2_SO_4_ after 10 min. The extinction was measured at 450 nm (reference wavelength 620 nm).

### Adaptation of a commercial CCHFV IgG-IFA

A commercially available IFA (Euroimmun) for the detection of CCHFV-specific antibodies in human sera was adapted for use in cattle. The assay based on transfected cells expressing the CCHFV Gc- and cells expressing the N-protein. For the detection of antibodies in serum samples from cattle, all incubation steps were performed for 30 min at 37°C and the slides were washed for 5 min in PBS containing 0.1% Tween20. Bovine sera were diluted 1:50 in TBST buffer (0.05 M Tris; 0.138 M NaCl; 0.0027 M KCl; 0.1% Tween20; pH 10) and 25 μl of this dilution was used for each well on the slide. After washing as described above, 20 μl of goat anti-bovine IgG-FITC conjugate (Southern Biotech, Birmingham, Alabama, USA) diluted 1:40 in TBST (containing 0.002% Evans Blue) was added to each well. Following another identical washing step, glycerin was added and the results were visualized with a fluorescence microscope.

### Development of a new CCHFV IgG-ELISA

The new ELISA based on a His-tagged recombinant Nucleocapsid (N-) protein of the CCHFV strain Kosovo Hoti (Accession no. DQ133507), which was expressed in *E*. *coli* and purified by Nickel-chelate chromatography. The incubation steps were performed in an incubator with 5% CO_2_. Wells were coated with 0.2 μg antigen in 100 μl coating buffer (PBS; 1% BSA; pH 11) for 1 h at 37°C and afterwards blocked with 200 μl/well blocking buffer (IDVet, Grabels, France) for 1 h at 37°C. Sera, diluted (1:40) in serum dilution buffer (no. 11, IDVet, 100 μl/well) were added in duplicate to wells coated with antigen and without antigen and incubated for 1 h at 37°C. Plates were washed three times with PBS-Tween20 0.1% and bound antibodies were detected using goat anti-bovine IgG-HRP conjugate (Southern Biotech) diluted (1:2000) in conjugate dilution buffer (no. 3, IDVet, 100 μl/well) for 2 h at 37°C. After another washing step, 100 μl/well of TMB solution (Bio-Rad) was added and incubated for 20 min. The reaction was stopped with H_2_SO_4_ and measured at 450 nm against a reference wavelength of 620 nm. The result (R-sample) for each sample was obtained by subtracting the average OD value of the reactions without antigen from the reactions with antigen. The final result (fR) was given as a percentage of R-sample to the result of the positive control (R-positive) on each plate (fR = (R-sample / R-positive) * 100).

### Performance of seroepidemiological investigations

In general, the investigation of serum samples of the seroepidemiological study has been performed according to a flow-chart designed for such studies [16]. Briefly, all samples have been tested in the new as well as in the commercial ELISA to confirm the cut-off values and the results from the validation. All “positive” or “equivocal” results have been retested in the respective ELISA for confirmation. Serum samples with a positive result in one of the ELISAs or an equivocal result in both ELISAs which were afterwards positive in the IFA have been declared as positive.

### Accession numbers

DQ133507: CCHFV strain Kosovo Hoti, complete S segment

## Results

### Adaptation and validation of the commercial CCHFV IgG-ELISA

The VectorBest ELISA has frequently been used in studies on human sera in the past. We have adapted this assay for investigation of bovine sera and determined its specificity and sensitivity by comparative analysis with results of the also adapted commercial IFA (Euroimmun). Two cut-off values were defined for the VectorBest ELISA: 0.4 and 0.5. Samples giving OD values lower than 0.4 were classified as “negative”, samples with OD values higher than 0.5 as “positive”, and samples with values 0.4 or 0.5 as “equivocal”. The diagnostic sensitivity and specificity was calculated by testing 56 IFA positive serum samples from cattle from Turkey and 137 serum samples from cattle from Germany (Table 1). The samples from Germany were defined as negative, because CCHFV has never been found in this area of Europe.

**Table 1:** Results of the adapted commercial ELISA for serum samples from cattle.

		IFA (Euroimmun) positive	defined as negative by origin
ELISA (Vector-Best)	positive	55	0
	negative	1	137
	equivocal	0	0

Comparative analysis of the results of the adapted commercial ELISA and the adapted commercial IFA for serum samples from cattle. The negative serum samples from Germany have been defined as negative and have not been tested in the IFA.

All negative sera were “true negative” in the commercial ELISA and only one IFA positive serum was “false negative”. Based on these results the diagnostic sensitivity (D-SN) was determined as the ratio of true-positives (n = 55) / (true-positives (n = 55) + false-negatives (n = 1)), which leads to a result of 98%. The diagnostic specificity (D-SP) was calculated as the ratio of true-negatives (n = 137) / (true-negatives (n = 137) + false-positives (n = 0)) and resulted in a value of 100%. Within the confidence interval of 95% the allowed error for the D-SN was 4%, and for the D-SP 2% [17].

### Adaptation and validation of the new CCHFV IgG-ELISA

102 bovine sera originating from a CCHFV endemic area in Turkey were tested with the commercial ELISA and with the commercial IFA. 54 out of the 102 sera tested positive in both reference assays were used as positive samples and 503 sera from cattle from Germany were used as negative samples for the validation of the new ELISA (Table 2).

**Table 2:** Results of the new ELISA for serum samples from cattle.

		reference assays positive	defined as negative by origin
new ELISA	positive	52	3
	negative	1	498
	equivocal	1	2

Comparative analysis of the results of the new ELISA and the combined result of the adapted commercial reference assays (IFA and ELISA) for serum samples from cattle. The negative serum samples from Germany have been defined as negative and have not been tested in one of the reference assays.

52 sera from Turkey were correctly classified as “positive” by the new CCHFV ELISA, while one sample gave a “false negative” and one an “equivocal” result. Samples with a fR value below 15% were classified as “negative”, with a fR value higher than 25% as “positive” and samples with a fR value between as “equivocal”. 498 of 503 negative sera were classified correctly, while three sera were “false positive”.

The D-SN of the in-house ELISA was 98% and the D-SP was 99%. For 0.5% of the sera the status could not be determined. The allowed error for the D-SN was 4%, and for the D-SP 1% within the confidence interval of 95% [17].

### Seroepidemiological study

The new and the commercial ELISA were used in parallel as screening tests for investigating the CCHFV seroprevalence rate in cattle sera from the Former Yugoslav Republic of Macedonia. The results were nearly identical, except that the new ELISA gave a higher number of “equivocal” results (Table 3).

**Table 3:** Results of the different assays used in the seroepidemiological study.

Region	Result	New ELISA	ELISA (Vector-Best)	IFA (Euroimmun)	Final result
Northeastern Region	positive	15	16	16	16
	negative	3	4	-	4
	equivocal	2	0	-	0
Skopje Region	positive	2	3	3	3
	negative	32	32	-	32
	equivocal	1	0	-	0
Vardar Region	positive	3	3	4	4
	negative	19	19	-	19
	equivocal	1	1	-	0
Southeastern Region	positive	1	0	-	0
	negative	77	80	-	80
	equivocal	2	0	-	0

The numbers of positive, negative and equivocal results of each test are shown for every region. The final result is a combination of the results of the individual assays.

**Fig 1:**
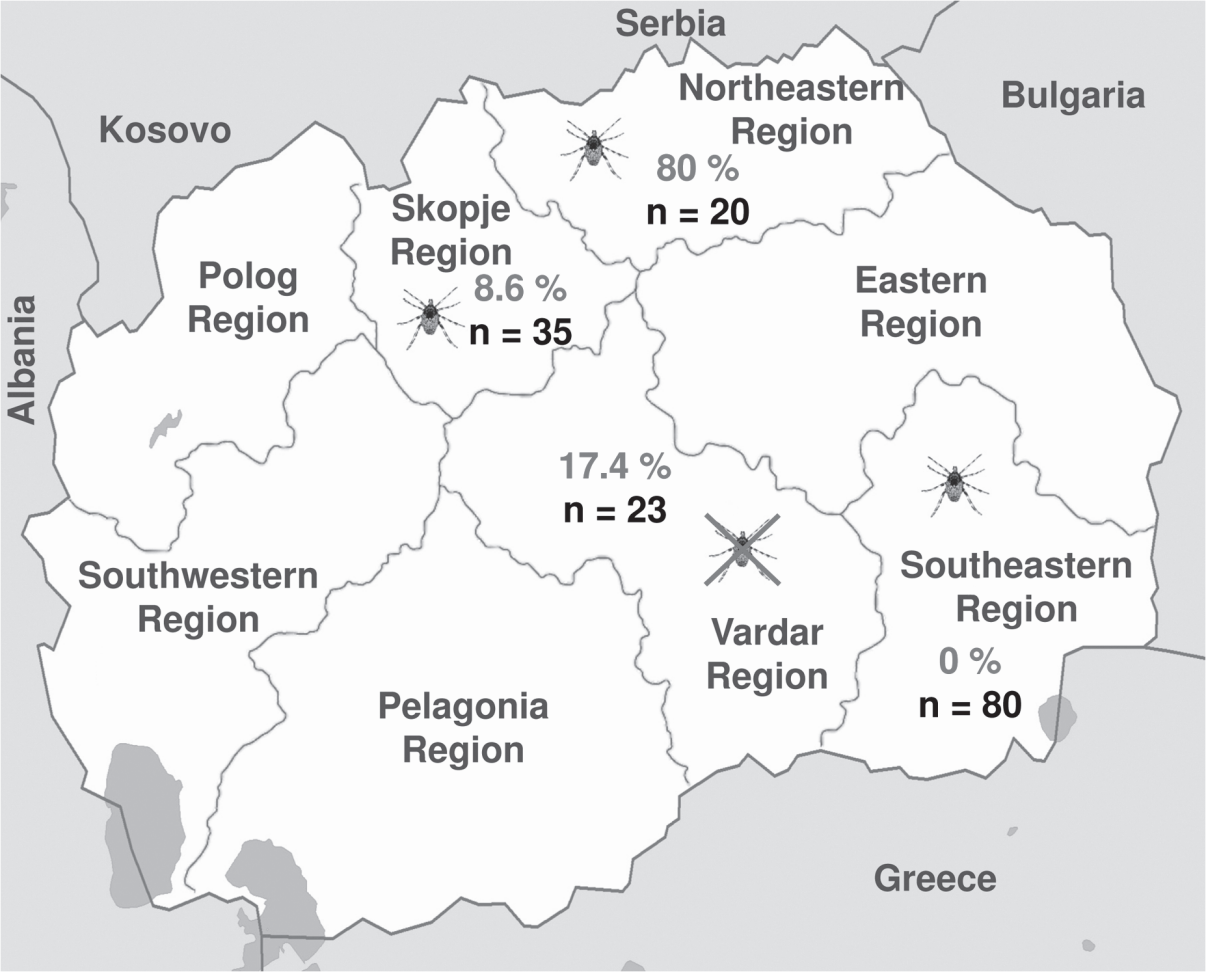
Seroprevalence in different regions of Former Yugoslav Republic of Macedonia. The map illustrates the prevalence of Crimean-Congo Hemorrhagic Fever Virus specific antibodies in cattle in four regions of the Former Yugoslav Republic of Macedonia. The ticks show in which regions Hyalomma ticks have been identified and the crossed out tick shows where no Hyalomma ticks have been identified. (Figure modified from Wikimedia Commons [18]).

Out of 158 serum samples 23 were confirmed positive (14.6%). Evaluated by geographical region, 16 sera (80%) from the Northeastern region, 3 (8.6%) from the Skopje region and 4 (17.4%) from the Vardar region were positive, while no serum sample was found positive in the Southeastern region. The results reveal great differences in the antibody prevalence among the four studied regions (Fig. 1) with the highest prevalence in the Northeastern region of the country.

### Collection of ticks

A total of 128 ticks were collected from cattle, sheep and goat. Overall 66% (n = 84) were males and 34% (n = 44) females. In the Northeastern region 23% (n = 13) of the collected ticks were females, in the Skopje region 100% (n = 7), in the Southeastern region 37% (n = 22), and in the Vardar region 50% (n = 2). The ratio of Hyalomma (n = 34) and Rhipicephalus (n = 94) was 1:3. *Hyalomma* ticks have been found in every of these regions except in the Vardar region (Table 4).

**Table 4:** Results of the tick collection.

Region	Tick species	Male	Female	Number of ticks
Northeastern Region	*Hyalomma marginatum*	12	3	15
	*Rhipicephalus bursa*	32	10	42
Skopje Region	*Hyalomma marginatum*	0	1	1
	*Rhipicephalus bursa*	0	6	6
Southeastern Region	*Hyalomma marginatum*	18	0	18
	*Rhipicephalus bursa*	15	13	28
	*Rhipicephalus annulatus*	5	9	14
Vardar Region	*Hyalomma marginatum*	0	0	0
	*Rhipicephalus bursa*	2	2	4

The table shows the collected number of ticks of the genus *Hyalomma* and *Rhipicephalus* for the different regions. The overall number of ticks is shown as well as the number of males and females.

## Discussion

Here we describe the adaptation of two commercial assays and the development of a novel ELISA for CCHFV IgG antibody testing of bovine sera. Assays were adjusted to give diagnostic sensitivities and specificities of more than 98%.

As a proof of principle, cattle sera from the Former Yugoslav Republic of Macedonia were tested in the new ELISA as well as in the commercial ELISA and positive results were reconfirmed in the IFA. The results demonstrate the stability and the high performance of the novel assay.

The seroepidemiological study provides the first evidence for the circulation of CCHFV in Former Yugoslav Republic of Macedonia. This finding was not unexpected since Former Yugoslav Republic of Macedonia has, next to Serbia and Greece, a border to Kosovo, Albania, and Bulgaria, where many human cases occurred over the last years/decades [9]. In case of Former Yugoslav Republic of Macedonia only a few human CCHF cases have ever been notified and none has been published. Therefore, the high antibody prevalence of 80% in cattle from the Northeastern region is indeed surprising, and close to the antibody prevalence in high endemic areas in Turkey. This indicates that the risk for human CCHFV infections in the Northeastern region is quite high. In the Central region of the country the prevalence is less but significant, particular since no CCHFV-specific antibodies were detected in cattle from the Southeastern region.

In addition to the detection of CCHFV-specific antibodies, the present paper demonstrates the occurrence of the main vector, namely ticks of the genus *Hyalomma*, in Former Yugoslav Republic of Macedonia for the first time. In this study, a correlation could not be observed between the ratio of Hyalomma ticks and Rhipicephalus ticks and the antibody prevalence in the respective regions. In the Northeastern region the rate of Hyalomma ticks was 26% (n = 15), but the antibody prevalence was the highest (80%). In the Southeastern region the percentage of Hyalomma ticks was 30% (n = 18) and no CCHFV-specific antibodies were detected. Next to the juvenile stages of the ticks, only females need a blood meal and therefore only those are involved in the infection cycle. Focusing only on ticks of the genus *Hyalomma*, the results of the study might indicate that a higher percentage of females results in a higher prevalence of CCHFV-specific antibodies. In the Northeastern region the percentage of females was 20% (n = 3/15) and in the Southeastern region 0% (n = 0/18). The respective antibody prevalence was 80% and 0%. In the other regions the number of collected Hyalomma ticks was too small to be included. However, a correlation could not be observed between the general ratio of the tick gender (Hyalomma and Rhipicephalus) and the antibody prevalence in the respective regions. In the region with the lowest rate of female ticks (Northeastern region, 23%), the highest prevalence was detected (80%). This would highlight the importance of Hyalomma ticks for the infection cycle of CCHFV [1]. On the other hand, no Hyalomma ticks were found in the Vardar region, but an antibody prevalence of 17.4% was determined. For further analyses it would be necessary to collect more ticks from different host species over a longer time period. However, the differences in the CCHFV antibody prevalence in cattle may be also due to a variable prevalence of CCHFV in the tick populations, as well as differences in the animal husbandry and land use. To explain the infection cycle in Former Yugoslav Republic of Macedonia much more information would be needed. Especially, an investigation of the CCHFV prevalence in the tick population would be important.

In any case it is mandatory to continue and intensify the collection of ticks and serum samples in all regions of Former Yugoslav Republic of Macedonia and to screen those for CCHFV and CCHFV-specific antibodies, respectively. The high prevalence indicates a high CCHFV infection rate in cattle. This and the frequency of outbreaks in neighboring countries conflicts with the extremely low number of registered human CCHF cases in the Former Yugoslav Republic of Macedonia. It seems to be important to improve the diagnostic capacity, as well as the awareness for this disease among physicians in the country. It may be considered to implement protection and management measures to reduce infection risks for humans [9].

## Supporting Information

S1 Checklist STARD checklist. Item #3: The samples were collected as part of the program for control and eradication of bovine brucellosis. No inclusion or exclusion criteria were set. Item #4: The recruitment was randomized, especially since CCHFV infected ruminants develop no clinical signs. Item #6: It is a prospective study. Item #8: All assays used were newly developed or adapted. The technical specifications are described on page 7. Item #10: Most of diagnostic work was performed at the NRL for CCHFV at the Friedrich-Loeffler-Institut. All persons were trained well. Item #11: The IFA was performed without having the information of previous results. Item #15: There is no further information available. Item #19: The comparison of the results of the different assays can be found in Table 3.
(DOC)
